# Effect of the crude extract of *Eugenia uniflora* in morphogenesis and secretion of hydrolytic enzymes in *Candida albicans* from the oral cavity of kidney transplant recipients

**DOI:** 10.1186/s12906-015-0522-x

**Published:** 2015-02-05

**Authors:** Walicyranison Plinio Silva-Rocha, Vitor Luiz de Brito Lemos, Magda Rhayanny Assunção Ferreira, Luiz Alberto Lira Soares, Terezinha Inês Estivalet Svidzisnki, Eveline Pipolo Milan, Guilherme Maranhão Chaves

**Affiliations:** Laboratório de Micologia Médica e Molecular, Departamento de Análises Clínicas e Toxicológicas, Universidade Federal do Rio Grande do Norte, Natal, Rio Grande do Norte Brazil; Departamento de Farmácia, Universidade Federal de Pernambuco, Recife, Pernambuco Brazil; Departamento de Análises Clínicas, Universidade Estadual de Maringá, Maringá, Paraná Brazil; Departamento de Infectologia, Universidade Federal do Rio Grande do Norte, Natal, Rio Grande do Norte Brazil

**Keywords:** *Candida albicans*, Oral candidiasis, Kidney transplant recipients, Morphogenesis, Hydrolytic enzymes, *Eugenia uniflora*

## Abstract

**Background:**

*Candida albicans* is a diploid yeast that in some circumstances may cause oral or oropharyngeal infections. Yeasts virulence factors contribute for both the maintenance of colonizing strains in addition to damage and cause tissue invasion, thus the establishment of infection occurs. The limited arsenal of antifungal drugs for the treatment of candidiasis turn the investigation of natural products mandatory for the discovery of new targets for antifungal drug development. Therefore, tropical countries emerge as important providers of natural products with potential antimicrobial activity. This study aimed to investigate morphogenesis and secretion of hydrolytic enzymes (phospholipase and proteinase) in the presence of the CE of *Eugenia uniflora*.

**Methods:**

The isolates were tested for their ability to form hyphae in both solid and liquid media under three different conditions: YPD + 20% FBS, Spider medium and GlcNac and the ability to secrete phospholipase and proteinase in the presence of 2000 μg/mL of *E. uniflora*.

**Results:**

The CE of *E. uniflora* inhibited hypha formation in both liquid and solid media tested. It also impaired hydrolytic enzymes production.

**Conclusions:**

This was the first study to describe the interaction of a natural product with the full expression of three different factors in *C. albicans. E. uniflora* may be an alternative therapeutic for oral candidiasis in the future.

## Background

*Candida* species are the most common human fungal pathogens, whereas *Candida albicans* is the most prevalent species isolated in fungal infections of the oral mucosa [1-3]. In the human oral cavity, these yeasts colonize from 20 to 80% of adults without evidences of infection [3-5]. However, in some circumstances which involve both pathogen attributes of virulence as well as host immunity; specific groups of patients, such as those with AIDS, transplant recipients and patients submitted to broad spectrum antibiotics may become susceptible to develop oropharyngeal candidiasis [6-8].

Oral candidiasis is a fungal opportunistic infection commonly observed in kidney transplant recipients, where the prevalence of the disease ranges from 9.4 to 46% in different group of patients, being *C. albicans* the most frequently isolated species [6,9-13].

*C. albicans* virulence factors contribute for both the maintenance of colonizing strains and tissue invasion, thus the establishment of infection occurs [14-16]. Among the main virulence attributes of *C. albicans* are the ability of cells transition from yeast to hyphae, a phenomenon called morphogenesis, which contributes to tissue invasion [17]. Morphogenesis is a well-recognized virulence factor in *C. albicans*, because mutants unable to form pseudohyphae or true hyphae are attenuated in virulence [18,19].

Merson-davies and Odds [20] proposed a quantitative index based on the shape of the cells to differentiate blastoconidia, pseudohyphal and true hyphal forms of growth. Yeast cells have a morphology index (MI) between 1.0 to 1.5, pseudo hyphae from 2.5 to 3.4 and true hyphae greater than 3.4 [21]. This index, based on a complex image analysis measurements can be substituted with an approximated score system to generate information on morphology distributions [22,23].

The secretion of hydrolytic enzymes such as phospholipases, which degrade glycerolphospholipids of cell membranes, is important for host cells destruction [24]. In addition, *C. albicans* possesses secreted aspartic proteinases (Saps), able to target several important substrates, such as albumin, immunoglobulins and extracellular matrix proteins [14,25,26].

The limited arsenal of antifungal drugs for the treatment of candidiasis includes mainly polyenes, azoles and echinocandins [27-29]. Nevertheless, these drugs have narrow therapeutic index, low bioavailability, week intestinal absorption and serious side effects [30]. In addition, the excessive and indiscriminate use of antifungal agents favors the development of resistant strains [31]. Although *C. albicans* has been reported as less resistant to antifungal drugs than other *Candida* species, resistance to azoles [32] and echinocandins [33] have been reported. Therefore, due to the increase of resistance of pathogenic fungi to antifungal drugs currently used, there is a necessity for the prevention of oral fungal infections and the development of alternative therapies [34,35].

The use of natural products aiming the treatment of several diseases has increased in the last few years, related to the high diversity of plants distributed around the planet [36]. Nevertheless, there are only a few studies about the use of vegetal extracts to treat fungal infections, including candidiasis [37-39]. In addition, very little is known about the action of plant extractive compounds in the virulence attributes of *C. albicans.*

Concerning to the world biodiversity, the American flora is one of the most rich source of plants with pharmacological activities [40] In this context, Brazil emerges as an important provider of botanical material for the international pharmaceutical market [40]. *Eugenia uniflora*, (known in Brazil as “pitangueira”) is a native Brazilian plant belonging to the family *Myrtaceae*, which includes species that have phenolic compounds as the predominant constitution [41]. The infusion of *E. uniflora* leaves has application in popular medicine mainly due to its properties as antioxidant, hypotensive, modulator of antibiotics and antifungal drugs [42]. Actually, anti-*Candida* activity of *E. uniflora* has been previously described [42,43].

The present study aimed to evaluate the action of the crude extract (CE) of *E. uniflora* in some *C. albicans* virulence factors, including morphogenesis and hydrolytic enzymes (proteinase and phospholipase) activities of clinical oral isolates of *C. albicans* obtained from kidney transplant recipients in Brazil.

## Methods

### Plant material and *Eugenia uniflora* extract

*E. uniflora* leaves were collected in Ceará-Mirim city, in the east of Rio Grande do Norte state, Brazil. The plant was identified at the Herbarium of the Federal University of Rio Grande do Norte (Department of Botany, Ecology and Zoology, Biosciences Center), and a voucher specimen was deposited (n° 11763). The leaves were dried at room temperature and ground into powder. Subsequently, 10 g of the dried leaves powder was submitted to turbo extraction with 100 mL of acetone:water (7:3, v/v), in four rounds of five minutes. The resulting extract was filtered and concentrated by rotary evaporation at 40°C, 150 rpm (Laborota 4000, Heidolph). The concentrated was submitted to lyophilization at -64°C, 0.006 mBar (ALPHA 1-2 LDplus, Fisher Scientific, France) and the extract was stored at 4°C.

### Strains and culture conditions

We evaluated a total of 48 *C. albicans* clinical isolates obtained from the oral cavity of kidney transplant recipients, belonging to the Medical and Molecular Mycology Laboratory, Department of Clinical and Toxicological Analyses, Federal University of Rio Grande do Norte. Only patients who agreed to take part on a surveillance confidential study, in accordance to the Local Research Ethics committee from The Onofre Lopes University Hospital, approved under the number 152/07, were enrolled in this study. Two references strains, *C. albicans* ATCC90028 and *C. albicans* SC5313 were used as control organisms. All the strains were cultivated on the surface of Sabouraud Dextrose Agar (Dextrose 40 g, Peptone 10 g, Agar 15 g, Distilled water 1000 mL) and incubated at 37°C for 48 h before the experiments. The strains were stored in YPD broth (Dextrose 2 g, Peptone 2 g, Yeast extract 1 g, and Distilled Water, 100 mL) with 20% glycerol at -80°C.

### Inoculum standardization for *Candida albicans* virulence factors evaluated “*in vitro*”

For all the virulence factors evaluated *in vitro*, the samples were initially grown in NGY medium (Difco Neopeptone 1 g/L, Dextrose 4 g/L; Difco yeast extract 1 g/L). *C. albicans* cells were incubated for 18-24h in a rotatory shaker (Tecnal, TE-420, São Paulo, Brazil) at 30°C, 200 rpm. This culture medium produces an inoculum size of about 2 × 10^8^ cells/mL, spectrophotometrically measured at a wavelength of 600 nm (Biochrom Libra S21/S22 spectrophotometer; [22]). Subsequently, *C. albicans* cells were diluted to obtain the specific inoculums needed for each attribute of virulence test evaluated *in vitro*.

### Micromorphological analysis of *Candida albicans* cells grown in NGY broth in the presence of *Eugenia uniflora*

For comparative purposes, *C. albicans* cells grown in NGY broth supplemented with 2000 μg/ml of the CE of *E. uniflora* (as previously described) were observed under an optical microscope (CX21, Olympus, Japan) and the micromorphological aspect of the cells was observed at 400x magnification.

### Morphogenesis of *Candida albicans* in YPD broth added 20% fetal bovine serum, spider broth and n-acetyl-d-glucosamine broth (GlcNac)

The morphogenesis assay was performed according to the technique described by Chaves et al. [22]. *C. albicans* cells were standardized to 1×10^6^ cells/mL and subsequently inoculated into three distinct morphogenesis induction culture media: YPD broth added Fetal Bovine Serum (Sigma- Aldrich) at a concentration of 20%, Spider broth (nutrient broth 1 mL, mannitol 1 g, dibasic potassium phosphate 0.2 g, destilated water 99 mL, pH 7.5), and GlcNac broth (Yeast Nitrogen Base -Difco®- 6.7 g, N-acetyl-d-glucosamine-Himedia®- 20g, destilated water 1000 mL) and then incubated during 3 h at 37°C - 200 rpm in a rotatory shaker in the presence and absence of 2000 μg/ml of the CE of *E. uniflora*. After 3-h incubation, culture samples were mixed with an equal volume of 10% formaldehyde to arrest further development. The 3-h sample was examined to approximate the mean morphology index (MI), in which a value close to 1 indicates a population of spheroidal yeast cells and value close to 4 indicates a population of true hyphal cells. Values between 2.5 to 3.4 indicate pseudohyphal morphologies. One hundred cells of each strain were scored.

### Measurements of *Candida abicans* hyphal cells length

*C. albicans* cells length was measured after morphogenesis induction (3h incubation in YPD + 20% FBS). For this proposal, the Tsview software® (Eclipse E100, Nikon, Japan) was employed. For each strain, the mean of 100 hyphal cells length was determined for the isolates previously grown in the presence or the absence of *E. uniflora*.

### Morphogenesis of *Candida albicans* on solid media

For the induction of hyphal formation on solid media, the cells were grown in NGY, centrifuged and washed three times in water. From the cell suspensions, 5 μL was spotted on the surface of Spider medium (Nutrient agar 10 g, Mannitol 10 g, KH_2_PO_4_ 2 g, agar 14.5 g, Distilled water 1000 mL [44]) and GlcNac medium (Yeast Nitrogen Base -Difco®- 6,7 g, N-acetyl-d-glucosamine-Himedia®- 20 g, agar 20 g, destilated water 1000 mL) in the presence and absence of 2000 μg/ml of the CE of *E. uniflora*. Alternatively, cells were also grown on the surface of YPD + 20% FBS agar, as previously described for Spider Medium. The plates were incubated at 30°C for seven days for subsequent observation of macromorphological aspects of the colonies. The assay was performed in triplicate.

### *Candida albicans* phospholipase assay

Phospholipase activity was determined with the method of Price et al. [45]. NGY overnight cultures were standardized to an inoculum size of 2×10^5^ cells/ml, and five μL of the cell suspension was inoculated in triplicate on the surface of phospholipase agar (10 g peptone, 40 g dextrose, 16 g agar, Egg Yolk Emulsion (Fluka) was added 80 mL, Distilled water 1000 mL) in the presence and absence of 2000 μg/ml of the CE of *E. uniflora* (10 g peptone, 40 g dextrose, 16 g agar, Egg Yolk Emulsion 80 mL, CE of *E. uniflora* 2000 μg/mL, Distilled water 1000 mL). The plates were incubated for 72 h at 30°C. After the incubation period, the precipitation zone (Pz) of the colonies was determined, with the formula described below:

$$ \mathrm{P}\mathrm{z} = \frac{\mathrm{Colony}\ \mathrm{diameter}\ \left(\mathrm{cm}\right)}{\mathrm{Colony}\ \mathrm{diameter}\ \left(\mathrm{cm}\right) + \mathrm{P}\mathrm{recipitation}\ \mathrm{z}\mathrm{one}\ \left(\mathrm{cm}\right)} $$

### *Candida albicans* proteinase assay

Proteinase activity was determined in the same manner as described for phospholipase activity, except by the fact that cells were inoculated on the surface of proteinase agar (11,7 g Yeast Carbon Base, 0,1 g Yeast Extract, 2 g Bovine Serum Albumine, CE of *E. uniflora* 2000 μg/mL, 16 g Agar, Distilled water 800 mL).

### Statistical analysis

Data were analyzed using the statistical software “Graph- Pad”, version 3.0. Results were presented as mean ± standard deviation, and differences were analyzed by the Mann–Whitney test. For all the analyses, P was considered a default value of 0.05 and the confidence interval of 95%.

## Results

### Micromorphological analysis of *Candida albicans* grown in the presence of *Eugenia uniflora*

**Figure 1:**
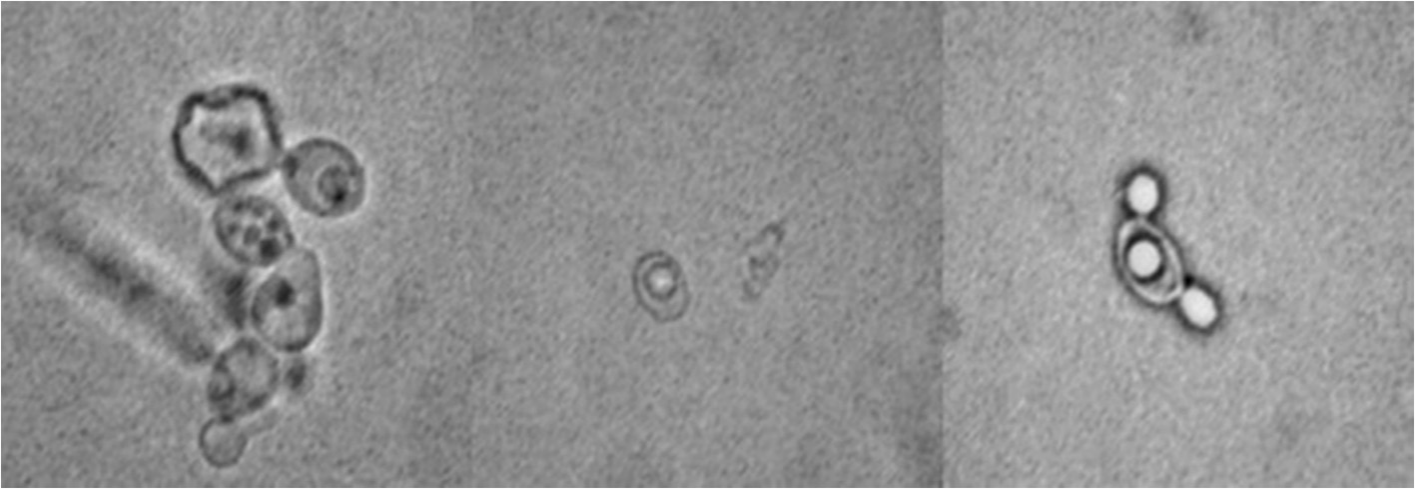
***Candida albicans*** **SC5314 pre-cultivated in NGY broth in the presence of 2000 μg/ml of the CE of** ***E. uniflora*** **for 24 h, 200 rpm, 30°C of incubation.** Blastoconidia show cell wall damage and large vacuoles (400x magnification).

The *C. albicans* reference strains SC5314 and ATCC90028 were pre-cultivated in NGY broth overnight at 30°C added 2000 μg/ml of *E. uniflora* and subsequently analyzed with optical microscopy. It was observed that the typical oval morphological form of *C. albicans* blastoconidia was altered; showing invaginations of the cell wall and an increase in the size of citoplasmatic vacuoles with this concentration of the CE (Figure 1). Therefore 2000 μg/ml was the concentration used to grow *C. albicans* cells to standardize the inoculum before all the experiments were performed. Furthermore, we adjusted the inoculum in a manner that both control (in the absence of *E.uniflora*) and test (in the presence of *E. uniflora*) experiments had the same amount of viable cells (determined by colony forming units counts after plating the cells on SDA, after 48 h incubation).

### *Candida albicans* hyphal induction in liquid medium

**Figure 2:**
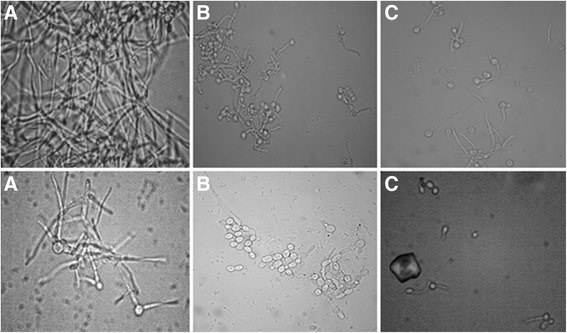
***Candida albicans*** **111R, a highly filamentous strain obtained from the oral cavity of a kidney transplant recipient after 3 h of incubation in YPD + 20% FBS (A), Spider (B) and GlcNac (C) liquid media at 37°C, 200 rpm.** Cells were either pre-cultivated in NGY broth in the absence (control experiments; lane 1) or presence of 2000 μg/ml (test experiments; lane 2) of the CE of *Eugenia uniflora* for 24 h, 200 rpm, 30°C of incubation, previously to the filamentation induction experiments.

The 48 clinical isolates of *C. albicans* and the reference strains ATCC90028 and SC5314 were tested for the ability to form hyphae in liquid YPD + 20% FBS, Spider and GlcNac broth after pre-cultivation of the cells in presence and absence of the CE of *E. uniflora.* When *C. albicans* cells were inoculated in liquid YPD + 20% FBS, the predominant morphology of the cells (previously grown in the absence of the CE) was pseudo-hyphae, with a mean MI ranging from 1.71 (strain 61) to 4.0 (strain 111R), after hyphal induction (Table 1). However, for the majority of the strains we observed a reduction in filamentation when cells were pre-cultivated in the presence of de CE. The mean MI was reduced in 41 of 50 strains analyzed (82%). The most notorious inhibition was observed for strain 17. The mean MI of 3.38 in the absence of the CE decreased to 1.82 when the cells were pre-cultivated in the presence of the CE of *E. uniflora*. In addition, *C. albicans* 111R, a highly filamentous strain found in our study (Mean MI = 4 in the absence of the CE of *E. uniflora*), also had a reduction in the mean MI in the presence of the CE (MI = 3.11; Table 1; Figure 2).

**Table 1:** **Mean morphology index (MI) of** ***Candida albicans*** **clinical isolates obtained from the oral cavity of kidney transplant recipients after 3 h of incubation in liquid media at 37°C, 200 rpm**

	**YPD+ 20% Fetal Bovine Serum**	**Spider broth**	**N-acetyl-D-glucosamine broth**
**Strain**	Absence of CE vs. Presence of CE*	Absence of CE vs. Presence of CE*	Absence of CE vs. Presence of CE*
**ATCC90028**	3.82 ± 0.50 vs. 2.47 ± 0.69	2.75 ± 1.46 vs. 1.03 ± 0.30	2.72 ± 0.96 vs. 2.08 ± 0.42
**SC5314**	3.28 ± 0.81 vs. 2.19 ± 0.68	3.52 ± 1.09 vs. 1.88 ± 1.37	2.59 ± 0.83 vs. 2.02 ± 0.79
**Strain01**	2.46 ± 0.70 vs. 3 ± 0.74	3.03 ± 0.95 vs. 1.38 ± 0.92	2.69 ± 0.81 vs. 2.14 ± 0.67
**Strain02**	3.43 ± 0.77 vs. 2.71 ± 0.87	2.9 ± 1.34 vs. 1.95 ± 0.76	2.06 ± 0.28 vs. 2.24 ± 0.57
**Strain03**	3.22 ± 0.64 vs. 2.47 ± 0.69	2.25 ± 0.66 vs. 0.95 ± 0.66	2.62 ± 0.74 vs. 2.28 ± 0.62
**Strain05**	2.23 ± 0.57 vs. 2.51 ± 0.59	2.75 ± 1.24 vs. 1.36 ± 0.88	2.09 ± 0.44 vs. 1.92 ± 0.49
**Strain06**	2.24 ± 0.51 vs. 2.08 ± 0.27	3.29 ± 1.12 vs. 1.39 ± 0.69	1.93 ± 0.33 vs. 1.91 ± 0.45
**Strain08**	1.86 ± 0.43 vs. 2 ± 0	3.57 ± 1.01 vs. 2.27 ± 1.46	2.01 ± 0.10 vs. 1.8 ± 0.40
**Strain10**	2.17 ± 0.62 vs. 2.7 ± 0.7	2.69 ± 1.36 vs. 1.69 ± 1.22	2.1 ± 0.54 vs. 1.98 ± 0.45
**Strain11**	2.15 ± 0.77 vs. 2.41 ± 0.57	3.62 ± 0.83 vs. 1.05 ± 0.51	2.49 ± 0.73 vs. 2.09 ± 0.57
**Strain12**	2.46 ± 0.56 vs. 2.32 ± 0.55	2.2 ± 0.74 vs. 1.11 ± 0.53	2.48 ± 0.61 vs. 2.03 ± 0.50
**Strain13**	2.17 ± 0.65 vs. 2.33 ± 0.57	2.99 ± 1.18 vs. 1.46 ± 0.99	2.26 ± 0.75 vs. 2 ± 0.59
**Strain17**	3.38 ± 0.63 vs. 1.82 ± 0.81	2.45 ± 0.83 vs. 1.24 ± 0.67	2.69 ± 0.79 vs. 2.34 ± 0.45
**Strain20**	3.7 ± 0.81 vs. 2.33 ± 0.57	3 ± 0.99 vs. 1.12 ± 0.52	3.55 ± 0.74 vs. 2.89 ± 0.85
**Strain21**	2.7 ± 0.92 vs. 2.58 ± 0.85	2.63 ± 1.28 vs. 1.57 ± 1.15	3.67 ± 0.53 vs. 3.21 ± 0.78
**Strain23**	2.8 ± 0.67 vs. 2.73 ± 0.61	2.07 ± 0.48 vs. 1.12 ± 0.59	2.3 ± 0.59 vs. 1.88 ± 0.52
**Strain24**	2.81 ± 0.74 vs. 1.8 ± 0.47	2.12 ± 0.54 vs. 1.25 ± 0.44	3.3 ± 0.78 vs. 2.73 ± 0.98
**Strain28**	3.24 ± 0.80 vs. 2.28 ± 0.53	1.64 ± 0.94 vs. 1.31 ± 0.90	3.54 ± 0.63 vs. 3.1 ± 0.87
**Strain30**	2 ± 0.62 vs. 2.32 ± 0.60	2.21 ± 0.46 vs. 1.1 ± 0.36	3.59 ± 0.62 vs. 3.25 ± 0.97
**Strain31**	2.79 ± 0.82 vs. 2.41 ± 0.48	2.2 ± 0.53 vs. 1.57 ± 0.70	3.63 ± 0.71 vs. 2.99 ± 0.82
**Strain32**	1.8 ± 0.65 vs. 2.2 ± 0.52	3.85 ± 0.48 vs. 2.33 ± 0.68	3.68 ± 0.49 vs. 3.1 ± 0.73
**Strain34**	2.15 ± 0.64 vs. 2.05 ± 0.50	2.6 ± 0.67 vs. 1.22 ± 0.52	3.57 ± 0.71 vs. 3.28 ± 0.74
**Strain37**	3.63 ± 0.80 vs. 2.87 ± 0.44	2.83 ± 0.79 vs. 1.2 ± 0.40	3.06 ± 0.83 vs. 3.36 ± 0.80
**Strain40**	2.85 ± 0.56 vs. 2.34 ± 0.68	1.98 ± 0.14 vs. 1.92 ± 0.27	3.79 ± 0.48 vs. 3.39 ± 0.65
**Strain41**	2.78 ± 0.69 vs. 2.3 ± 0.71	2.25 ± 0.52 vs. 1.79 ± 0.52	3.48 ± 0.77 vs. 2.99 ± 0.85
**Strain44**	2.45 ± 0.77 vs. 2.36 ± 0.83	2.07 ± 0.26 vs. 1.46 ± 0.50	2.15 ± 0.36 vs. 1.19 ± 0.61
**Strain46**	3.05 ± 0.77 vs. 2.23 ± 0.45	2.32 ± 0.57 vs. 1.52 ± 0.70	2.16 ± 0.47 vs. 1.96 ± 0.57
**Strain50**	2.84 ± 0.88 vs. 2.31 ± 1.01	2.99 ± 0.72 vs. 1.3 ± 0.46	2.49 ± 0.67 vs. 1.35 ± 0.70
**Strain51**	2.17 ± 0.43 vs. 2.14 ± 0.53	2.21 ± 0.46 vs. 1.2 ± 0.49	3.88 ± 0.38 vs. 1.41 ± 0.84
**Strain53**	2.64 ± 0.71 vs. 2.49 ± 0.67	2.36 ± 0.33 vs. 1.43 ± 0.73	2.07 ± 0.38 vs. 1.92 ± 0.42
**Strain54**	2.81 ± 0.40 vs. 2.34 ± 0.45	2.88 ± 0.82 vs. 1.6 ± 0.49	2.23 ± 0.51 vs. 1.19 ± 0.49
**Strain60**	2.36 ± 0.36 vs. 2.22 ± 0.26	1.88 ± 0.33 vs. 1.29 ± 0.67	2.41 ± 0.49 vs. 1.06 ± 0.24
**Strain61**	2.48 ± 0.75 vs. 1.71 ± 0.48	2.1 ± 0.39 vs. 1.08 ± 0.26	2.42 ± 0.61 vs. 1.05 ± 0.22
**Strain70**	2.99 ± 0.50 vs. 2.37 ± 0.49	2.54 ± 0.77 vs. 1.63 ± 1.05	2.53 ± 0.61 vs.1.21 ± 0.41
**Strain72**	2.31 ± 0.61 vs. 2.27 ± 0.51	2.37 ± 0.73 vs. 1.33 ± 0.70	2.72 ± 0.65 vs. 1.44 ± 0.69
**Strain82**	3.36 ± 0.93 vs. 2.62 ± 0.60	2.39 ± 0.58 vs. 1.31 ± 0.65	3.8 ± 0.60 vs. 1.47 ± 1.02
**Strain85**	3.15 ± 0.83 vs. 2.25 ± 0.63	1.98 ± 0.14 vs. 1.06 ± 0.31	2.77 ± 0.68 vs. 1.4 ± 0.53
**Strain107**	2.39 ± 0.63 vs. 2.19 ± 0.39	2.07 ± 0.33 vs. 1.06 ± 0.34	2.08 ± 0.31 vs. 1.27 ± 0.45
**Strain06A**	2.34 ± 0.71 vs. 2.1 ± 0.58	2.38 ± 0.68 vs. 1.6 ± 0.83	2.45 ± 0.59 vs. 1.53 ± 0.63
**Strain06L**	2.94 ± 0.58 vs. 2.56 ± 1.01	2.28 ± 0.59 vs. 1.52 ± 0.67	3.86 ± 0.45 vs. 1.13 ± 0.43
**Strain10Li**	2.42 ± 0.70 vs. 2.29 ± 0.62	2.01 ± 0.17 vs. 1.03 ± 0.17	2.42 ± 0.74 vs. 1.07 ± 0.29
**Strain10S**	2.54 ± 0.76vs. 2.65 ± 0.69	2.06 ± 0.55 vs. 1.02 ± 0.14	2.55 ± 0.86 vs. 1.12 ± 0.46
**Strain12A**	3.3 ± 0.79 vs. 2.44 ± 0.80	2.04 ± 0.20 vs. 1.09 ± 0.32	2.62 ± 0.74 vs. 1.16 ± 0.44
**Strain12L**	3.2 ± 0.68 vs. 2.31 ± 0.63	1.96 ± 0.20 vs. 1.09 ± 0.29	2.43 ± 0.59 vs. 1.19 ± 0.49
**Strain15A**	2.96 ± 0.83 vs. 2.38 ± 0.84	2.21 ± 0.46 vs. 1.2 ± 0.49	3.12 ± 0.87 vs. 1.11 ± 0.35
**Strain15G**	3.13 ± 0.81 vs. 1.92 ± 0.76	1.85 ± 0.36 vs. 1.26 ± 0.61	3.63 ± 0.61 vs. 1.4 ± 0.82
**Strain15L**	2.58 ± 0.68 vs. 2.34 ± 0.76	2.85 ± 0.89 vs. 1.3 ± 0.90	3.03 ± 0.89 vs. 1.63 ± 0.61
**Strain18A**	3.05 ± 0.67 vs. 1.74 ± 0.52	2.3 ± 0.56 vs. 1.53 ± 0.67	2.47 ± 0.64 vs. 1.97 ± 0.72
**Strain111L**	2.6 ± 0.70 vs. 2.36 ± 0.54	1.92 ± 0.42 vs. 1.26 ± 0.58	3.12 ± 0.57 vs. 1.33 ± 0.59
**Strain111R**	4 ± 0 vs. 3.11 ± 0.89	2.21 ± 0.46 vs. 1.21 ± 0.50	3.83 ± 0.53 vs. 3.55 ± 0.74

*CE: Crude Extract of Eugenia uniflora (2000 μg/mL).

Cells were either pre-cultivated in NGY broth in the absence (control experiments) or presence of 2000 μg/ml (test experiments) of the CE of *Eugenia uniflora* for 24 h, 200 rpm, 30°C of incubation, previously to the filamentation induction experiments. One hundred cells of each strain were scored.

The same trend was also observed when *C. albicans* strains were inoculated in Spider broth. The mean MI ranged from 1.64 (strain 61) to 3.85 (strains 111R), with an average of 2.47 for all the strains. However, a reduction in the morphology index was observed for all the isolates when cells were pre-cultivated with the CE of *E. uniflora* (mean of 1.37). The most significant reduction was observed for strain 11, which reduced the mean MI from 3.62 to 1.05 (reduction of 71%). The isolate 111R, a highly hyphae forming strain, had a reduction of the mean MI from 2.21 to 1.21 (45.3%; Table 1; Figure 2).

A possible reduction of the MI was also investigated in the hyphal inducing medium GlcNac broth. The mean MI ranged from 1.93 (strain 06) to 3.88 (strain 51), with an average of 2.82 and the prevalence of short pseudo-hyphae. When the isolates were pre-cultivated in the presence of the CE of *E. uniflora,* a reduction in the mean MI was observed for 48 of the 50 strains evaluated, with a mean MI of 1.98. The mean MI of the isolate 06 L was markedly reduced when the strain was pre-cultivated in the presence of CE of *E. uniflora* (3.86 to 1.13; 70.7%; Table 1). The mean MI for the strain 111R (highly filamentous) was also reduced in the presence of GlcNac. However, the reduction was not as expressive as when YPD + 20% FBS and Spider broth were used as hyphal inducing media (Table 1; Figure 2).

### *Candida albicans* measurments of hyphae lengths

Despite the fact that we could not observe a reduction in the mean MI for 18% of the strains tested when YPD + 20% FBS was used for hyphal induction, we noticed that although they were able to form true hyphae, hyphal sizes were visually shorter. In order to investigate the degree of reduction of the hyphae lengths, we randomly selected 13 isolates with low, moderate and high mean MI and the two reference strains after hyphal induction in YPD + 20% FBS with cells previously grown in the presence of the CE of *E. uniflora*. Hyphal sizes were observed with optic microscopy. We observed a reduction in hyphal size for all the 16 strains evaluated (including SC5314 and ATCC90028 reference strains). While the mean hyphal size was 21μm for the cells pre-cultivated in the absence of the CE, there was a reduction to 14 μm in the presence of the CE (Table 2). Hyphal size measurements were not performed with Spider or GlcNac broth because we could not visually observe length differences when several cells of each strain were observed in different microscopy fields.

**Table 2:** **Measurements** ***Candida albicans*** **hyphal sizes of clinical isolates obtained from the oral cavity of kidney transplant recipients after 3 h of incubation in liquid media at 37°C, 200 rpm**

**Hyphal size (μm)**	
**Strain**	Abscence of CE** vs. Presence of CE**
**ATCC 90028**	24.68 (24.68 ± 1.79) vs. 13.35 (13.35 ± 0.92)*
**SC5314**	30 (30 ± 2.47) vs. 16 (16 ± 1.16)*
**Strain 01**	21.67 (21.67 ± 1.39) vs. 15.02 (15.02 ± 1. 28)*
**Strain 05**	22.84 (22.84 ± 1.68) vs. 18.17 (18.17 ± 2.12)
**Strain 06**	19.3 (19.3 ± 1.23) vs. 11.24 (11.24 ± 1.79)*
**Strain 12**	10.16 (10.3 ± 1.89) vs. 16.62 (16.62 ± 1.34)
**Strain 23**	16.81 (16.81 ± 2.12) vs. 11.08 (11.08 ± 0,76)
**Strain 30**	10.06 (10.06 ± 0.86) vs. 14.32 (14.32 ± 0.97)
**Strain 37**	23.21 (23.21 ± 1.83) vs. 18.55 (18.55 ± 1. 48)*
**Strain 40**	23.28 (23.28 ± 1.94) vs. 13.6 (13.6 ± 0.91)*
**Strain 54**	15.35 (15.35 ± 1.41) vs. 3 (3 ± 0.32)*
**Strain 10Li**	21.25 (21.25 ± 1.87) vs. 11.73 (11.73 ± 0.92)*
**Strain 111R**	36.15 (36.15 ± 2.56) vs. 25.06 (25.06 ± 1.34)*

*P < 0.05, **CE: Crude Extract of Eugenia uniflora (2000 μg/mL).

Cells were either pre-cultivated in NGY broth in the absence (control experiments) or presence of 2000 μg/ml (test experiments) of the CE of *Eugenia uniflora* for 24 h, 200 rpm, 30°C of incubation, previously to the filamentation induction experiments. One hundred cells of each strain were scored.

### *Candida albicans* hyphal induction on solid media

**Figure 3:**
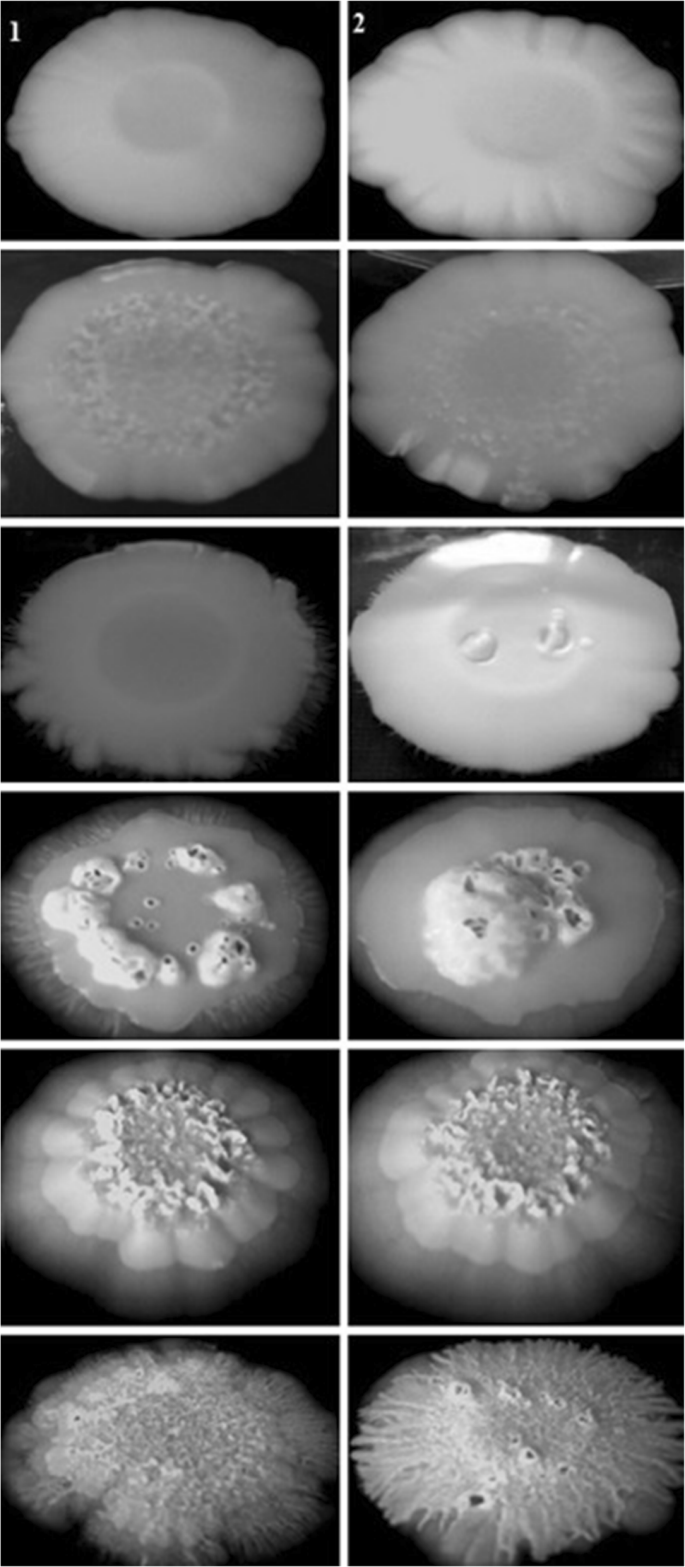
**Different phenotypes of hypha formation on YPD + 20% FBS agar of** ***Candida albicans*** **clinical isolates obtained from the oral cavity of kidney transplant recipients.** The isolates were incubated in triplicates at 30°C for seven days in the absence (control experiments; column 1) or presence of 2000 μg/ml (test experiments; column 2) of the CE of *Eugenia uniflora*.

**Figure 4:**
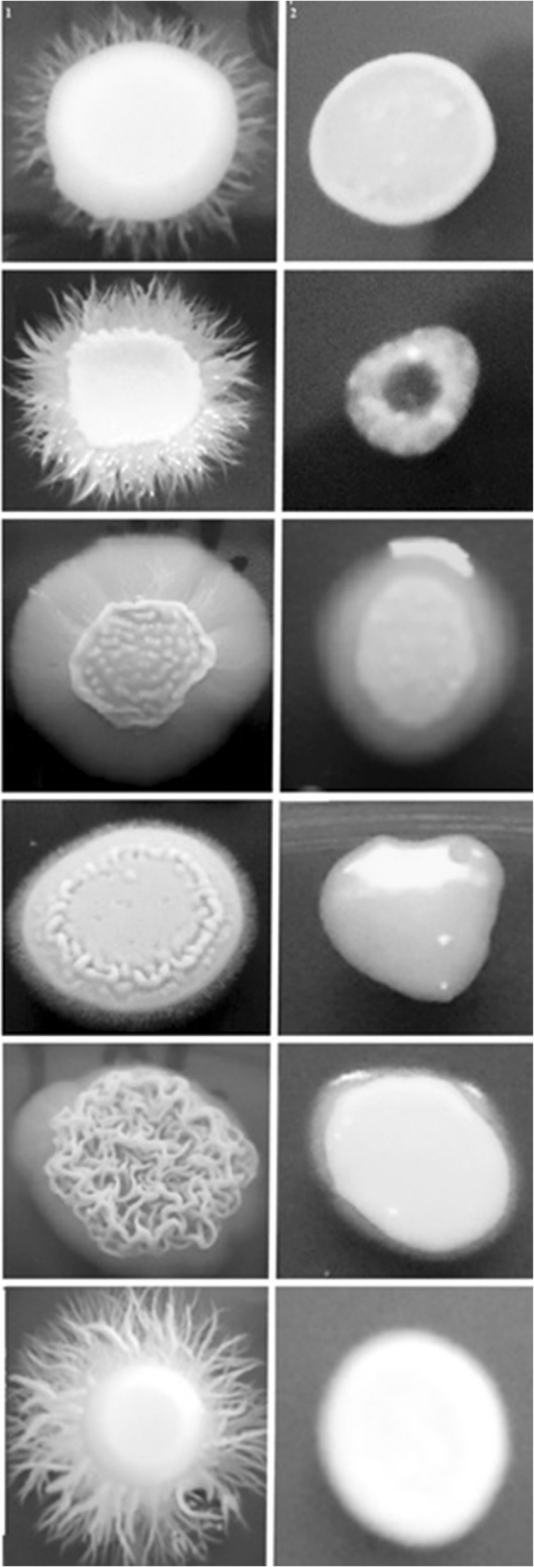
**Different phenotypes of hypha formation on Spider medium of** ***Candida albicans*** **clinical isolates obtained from the oral cavity of kidney transplant recipients.** The isolates were incubated in triplicates at 30°C for seven days in the absence (control experiments; column 1) or presence of 2000 μg/ml (test experiments; column 2) of the CE of *Eugenia uniflora.*

**Figure 5:**
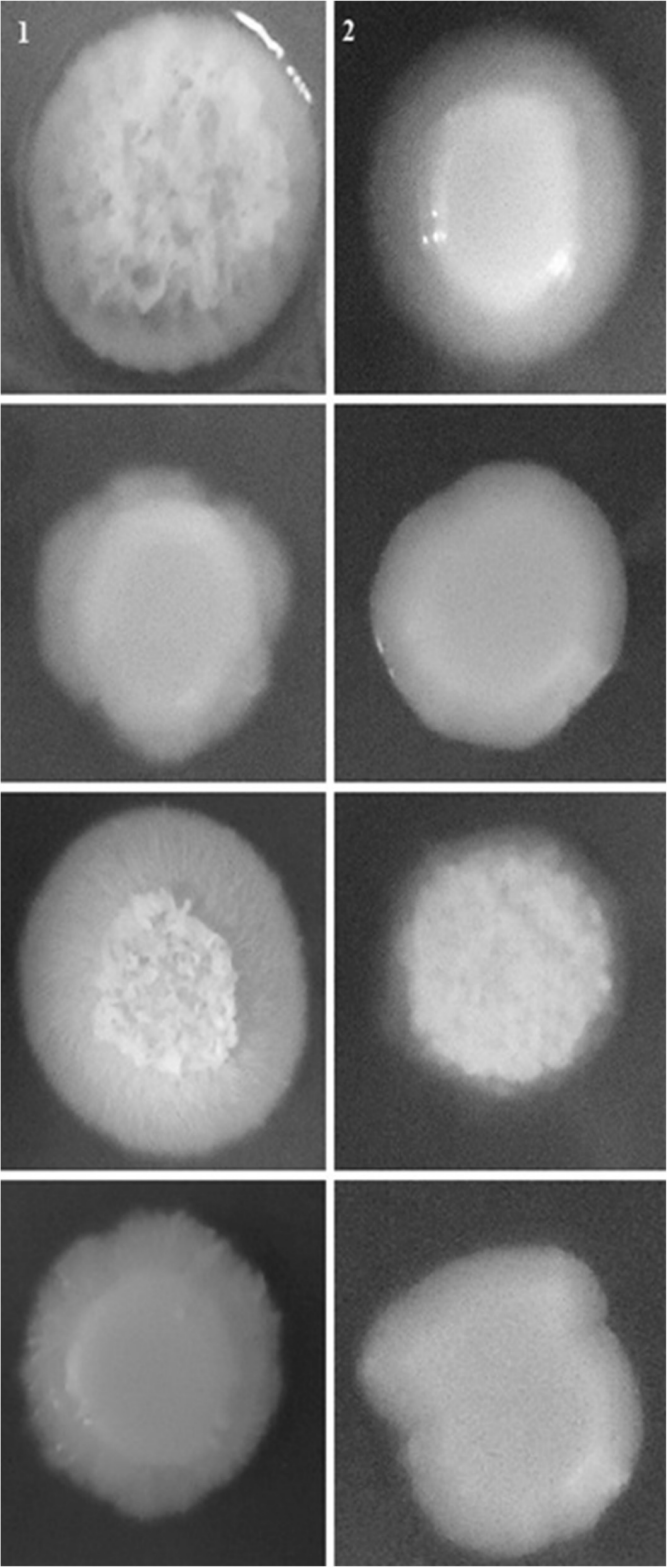
**Different phenotypes of hypha formation on N-acetyl-D-glucosamine of** ***Candida albicans*** **clinical isolates obtained from the oral cavity of kidney transplant recipients.** The isolates were incubated in triplicates at 30°C for seven days in the absence (control experiments; column 1) or presence of 2000 μg/ml (test experiments; column 2) of the crude extract of *Eugenia uniflora.*

We also evaluated a possible effect of the CE of *E. uniflora* in *C. albicans* hypha formation on solid media by cultivating all the 48 isolates and the two reference strains for seven days on YPD + 20% FBS agar, Spider medium and GlcNac agar medium at 30°C. The macromorphological aspect of the colonies was observed and the strains were markedly smoother than the respective control, grown in the absence of the CE, which showed wrinkled colonies (Table 3; Figures 3, 4 and 5). Despite the fact that YPD agar added 20% FBS was not an efficient inducer of filamentation for most of the isolates tested, for the strains that showed a wrinkled phenotype grown in this condition, smooth colonies were also observed for the respective strain grown in the presence of the CE of *E. uniflora* (Figure 3).

**Table 3:** **Hypha formation on solid media of** ***Candida albicans*** **clinical isolates obtained from the oral cavity of kidney transplant recipients**

	**YPD+ 20% Fetal Bovine Serum**	**Spider medium**	**N-acetyl-D-glucoasmine medium**
**Strain**	Absence of CE* vs. Presence of CE*	Absence of CE* vs. Presence of CE*	Absence of CE* vs. Presence of CE*
**ATCC90028**	Smooth vs. Smooth	Wrinkled vs. Smooth	Wrinkled vs. Smooth
**SC5314**	Wrinkled vs. Smooth	Wrinkled vs. Smooth	Wrinkled vs. Wrinkled
**Strain01**	Smooth vs. Smooth	Wrinkled vs. Smooth	Smooth vs. Smooth
**Strain02**	Smooth vs. Smooth	Wrinkled vs. Smooth	Wrinkled vs. Smooth
**Strain03**	Smooth vs. Smooth	Wrinkled vs. Smooth	Wrinkled vs. Smooth
**Strain05**	Smooth vs. Smooth	Wrinkled vs. Smooth	Wrinkled vs. Smooth
**Strain06**	Smooth vs. Smooth	Wrinkled vs. Smooth	Wrinkled vs. Smooth
**Strain08**	Smooth vs. Smooth	Wrinkled vs. Smooth	Wrinkled vs. Smooth
**Strain10**	Smooth vs. Smooth	Wrinkled vs. Smooth	Wrinkled vs. Smooth
**Strain11**	Smooth vs. Smooth	Wrinkled vs. Smooth	Wrinkled vs. Smooth
**Strain12**	Smooth vs. Smooth	Wrinkled vs. Smooth	Wrinkled vs. Smooth
**Strain13**	Smooth vs. Smooth	Wrinkled vs. Smooth	Wrinkled vs. Smooth
**Strain17**	Smooth vs. Smooth	Wrinkled vs. Smooth	Wrinkled vs. Smooth
**Strain20**	Smooth vs. Smooth	Wrinkled vs. Smooth	Wrinkled vs. Smooth
**Strain21**	Smooth vs. Smooth	Wrinkled vs. Smooth	Wrinkled vs. Smooth
**Strain23**	Smooth vs. Smooth	Smooth vs. Smooth	Wrinkled vs. Smooth
**Strain24**	Smooth vs. Smooth	Wrinkled vs. Smooth	Wrinkled vs. Smooth
**Strain28**	Smooth vs. Smooth	Wrinkled vs. Smooth	Wrinkled vs. Smooth
**Strain30**	Smooth vs. Smooth	Smooth vs. Smooth	Wrinkled vs. Smooth
**Strain31**	Smooth vs. Smooth	Wrinkled vs. Smooth	Wrinkled vs. Smooth
**Strain32**	Smooth vs. Smooth	Wrinkled vs. Smooth	Wrinkled vs. Smooth
**Strain34**	Smooth vs. Smooth	Wrinkled vs. Smooth	Smooth vs. Smooth
**Strain37**	Smooth vs. Smooth	Wrinkled vs. Smooth	Smooth vs. Smooth
**Strain40**	Smooth vs. Smooth	Wrinkled vs. Smooth	Wrinkled vs. Smooth
**Strain41**	Smooth vs. Smooth	Wrinkled vs. Smooth	Wrinkled vs. Smooth
**Strain44**	Smooth vs. Smooth	Wrinkled vs. Smooth	Wrinkled vs. Smooth
**Strain46**	Smooth vs. Smooth	Wrinkled vs. Smooth	Wrinkled vs. Smooth
**Strain50**	Smooth vs. Smooth	Wrinkled vs. Smooth	Wrinkled vs. Smooth
**Strain51**	Smooth vs. Smooth	Wrinkled vs. Smooth	Wrinkled vs. Smooth
**Strain53**	Smooth vs. Smooth	Wrinkled vs. Smooth	Wrinkled vs. Smooth
**Strain54**	Smooth vs. Smooth	Wrinkled vs. Smooth	Smooth vs. Smooth
**Strain60**	Smooth vs. Smooth	Wrinkled vs. Smooth	Wrinkled vs. Wrinkled
**Strain61**	Smooth vs. Smooth	Wrinkled vs. Smooth	Wrinkled vs. Wrinkled
**Strain70**	Smooth vs. Smooth	Wrinkled vs. Smooth	Wrinkled vs. Smooth
**Strain72**	Smooth vs. Smooth	Smooth vs. Smooth	Wrinkled vs. Smooth
**Strain82**	Smooth vs. Smooth	Smooth vs. Smooth	Wrinkled vs. Smooth
**Strain85**	Smooth vs. Smooth	Wrinkled vs. Smooth	Wrinkled vs. Smooth
**Strain107**	Smooth vs. Smooth	Wrinkled vs. Smooth	Wrinkled vs. Smooth
**Strain06A**	Smooth vs. Smooth	Wrinkled vs. Smooth	Wrinkled vs. Wrinkled
**Strain06L**	Wrinkled vs. Smooth	Wrinkled vs. Smooth	Wrinkled vs. Wrinkled
**Strain10Li**	Smooth vs. Smooth	Wrinkled vs. Wrinkled	Wrinkled vs. Smooth
**Strain10S**	Smooth vs. Smooth	Wrinkled vs. Smooth	Wrinkled vs. Smooth
**Strain12A**	Smooth vs. Smooth	Wrinkled vs. Smooth	Wrinkled vs. Smooth
**Strain12L**	Smooth vs. Smooth	Wrinkled vs. Smooth	Wrinkled vs. Smooth
**Strain15A**	Smooth vs. Smooth	Wrinkled vs. Smooth	Wrinkled vs. Smooth
**Strain15G**	Wrinkled vs. Wrinkled	Wrinkled vs. Smooth	Wrinkled vs. Smooth
**Strain15L**	Wrinkled vs. Wrinkled	Wrinkled vs. Smooth	Wrinkled vs. Wrinkled
**Strain18A**	Smooth vs. Smooth	Wrinkled vs. Smooth	Wrinkled vs. Smooth
**Strain111L**	Smooth vs. Smooth	Smooth vs. Smooth	Smooth vs. Smooth
**Strain111R**	Wrinkled vs. Wrinkled	Wrinkled vs. Smooth	Wrinkled vs. Wrinkled

*CE: Crude Extract of Eugenia uniflora (2000 μg/mL).

The isolates were incubated in triplicates at 30°C for seven days on YPD + 20% FBS, Spider medium and N-acetyl-D-glucosamine agar in the absence (control experiments) or presence of 2000 μg/ml (test experiments) of the CE of *Eugenia uniflora.*

### Effect of CE of *Eugenia uniflora* in secretion of hydrolytic enzymes

**Figure 6:**
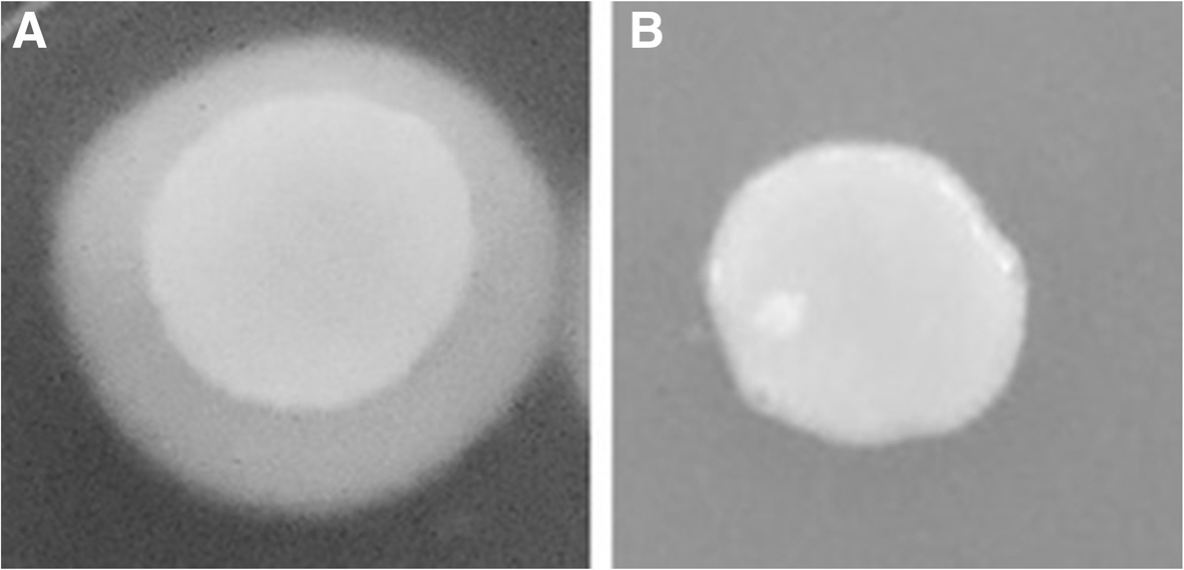
**Phospholipase activity on solid media of** ***Candida albicans*** **clinical isolates obtained from the oral cavity of kidney transplant recipients.** The isolates were incubated in triplicates at 30°C for 72 h on phospholipase agar in the absence (control experiments; **A)** or presence of 2000 μg/ml (test experiments; **B)** of the crude extract of *Eugenia uniflora*.

**Figure 7:**
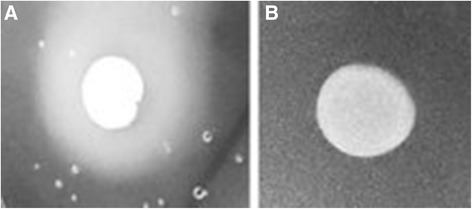
**Proteinase activity on solid media of** ***Candida albicans*** **clinical isolates obtained from the oral cavity of kidney transplant recipients.** The isolates were incubated in triplicates at 30°C for 72 h on proteinase agar in the absence (control experiments; **A)** or presence of 2000 μg/ml (test experiments; **B)** of the crude extract of *Eugenia uniflora*.

Other important virulence attribute of *C. albicans* is the ability to secrete hydrolytic enzymes. Therefore, we evaluated phospholipase and proteinase activity of the 48 clinical isolates of *C. albicans* and the references strains ATCC90028 and SC5314. When cells were grown on the surface of egg yolk emulsion agar (phospholipase induction), the average phospholipase zone values for all the 50 strains was 0.54 ± 0.18, considered a moderate activity (Table 4). When the CE of *E. uniflora* was included in the phospholipase media, the activity was completely inhibited (Figure 6). The same trend was observed for cells grown on the surface of the proteinase induction medium without the CE of *E. uniflora.* All the strains were able to produce satisfactory levels of proteinase (mean *Pz* value of 0.44 ± 0.21). A total inhibition of proteinase activity was observed for 48 out of 50 isolates of *C. albicans*, including the reference strains (Figure 7). In fact, only two strains (05 and 60; 4%) were still able to produce very low amounts of the hydrolytic enzyme (*Pz* of 0.88 ± 0.01 and 0.88 ± 0.00, respectively; Table 4).

**Table 4:** **Hydrolytic enzymes activity on solid media of** ***Candida albicans*** **clinical isolates obtained from the oral cavity of kidney transplant recipients**

	**Proteinase Activity (** ***Pz*** **†)**	**Phospholipase Activity (** ***Pz*** **†)**
**Strain**	Absence of CE** vs. Presence of CE**	Absence of CE** vs. Presence of CE**
**ATCC90028**	0.47 ± 0.03 vs. 1 ± 0.00*	0.80 ± 0.11 vs. 1 ± 0.00*
**SC5314**	0.59 ± 0.10 vs. 1 ± 0.00*	0.59 ± 0.04 vs. 1 ± 0.00*
**Strain01**	0.36 ± 0.04 vs. 1 ± 0.00*	0.55 ± 0.08 vs. 1 ± 0.00*
**Strain02**	0.51 ± 0.02 vs. 1 ± 0.00*	0.68 ± 0.02 vs. 1 ± 0.00*
**Strain03**	1 ± 0.00 vs. 1 ± 0.00	1 ± 0.00 vs. 1 ± 0.00
**Strain05**	0.29 ± 0.01 vs. 0.88 ± 0.01*	0.64 ± 0.00 vs. 1 ± 0.00*
**Strain06**	0.72 ± 0.11 vs. 1 ± 0.00*	0.60 ± 0.00 vs. 1 ± 0.00*
**Strain08**	0.72 ± 0.02 vs. 1 ± 0.00*	0.57 ± 0.00 vs. 1 ± 0.00*
**Strain10**	1 ± 0.00 vs. 1 ± 0.00	1 ± 0.00 vs. 1 ± 0.00
**Strain11**	0.43 ± 0.01 vs. 1 ± 0.00*	0.63 ± 0.04 vs. 1 ± 0.00*
**Strain12**	0.31 ± 0.02 vs. 1 ± 0.00*	0.75 ± 0.00 vs. 1 ± 0.00*
**Strain13**	0.69 ± 0.02 vs. 1 ± 0.00*	0.59 ± 0.01 vs. 1 ± 0.00*
**Strain17**	0.74 ± 0.02 vs. 1 ± 0.00*	0.50 ± 0.03 vs. 1 ± 0.00*
**Strain20**	0.61 ± 0.11 vs. 1 ± 0.00*	0.55 ± 0.03 vs. 1 ± 0.00*
**Strain21**	0.63 ± 0.02 vs. 1 ± 0.00*	0.58 ± 0.07 vs. 1 ± 0.00*
**Strain23**	0.65 ± 0.05 vs. 1 ± 0.00*	0.67 ± 0.04 vs. 1 ± 0.00*
**Strain24**	0.67 ± 0.12 vs. 1 ± 0.00*	0.49 ± 0.02 vs. 1 ± 0.00*
**Strain28**	0.44 ± 0.02 vs. 1 ± 0.00*	0.55 ± 0.05 vs. 1 ± 0.00*
**Strain30**	0.27 ± 0.05 vs. 1 ± 0.00*	0.68 ± 0.04 vs. 1 ± 0.00*
**Strain31**	0.47 ± 0.02 vs. 1 ± 0.00*	0.52 ± 0.02 vs. 1 ± 0.00*
**Strain32**	0.42 ± 0.04 vs. 1 ± 0.00*	0.75 ± 0.06 vs. 1 ± 0.00*
**Strain34**	0.75 ± 0.00 vs. 1 ± 0.00*	0.73 ± 0.03 vs. 1 ± 0.00*
**Strain37**	0.60 ± 0.06 vs. 1 ± 0.00*	0.50 ± 0.03 vs. 1 ± 0.00*
**Strain40**	0.53 ± 0.06 vs. 1 ± 0.00*	0.58 ± 0.05 vs. 1 ± 0.00*
**Strain41**	0.76 ± 0.10 vs. 1 ± 0.00*	0.55 ± 0.02 vs. 1 ± 0.00*
**Strain44**	1 ± 0.00 vs. 1 ± 0.00	0.64 ± 0.03 vs. 1 ± 0.00*
**Strain46**	0.57 ± 0.04 vs. 1 ± 0.00*	0.65 ± 0.02 vs. 1 ± 0.00*
**Strain50**	0.54 ± 0.03 vs. 1 ± 0.00*	0.63 ± 0.11 vs. 1 ± 0.00*
**Strain51**	0.39 ± 0.03 vs. 1 ± 0.00*	0.53 ± 0.00 vs. 1 ± 0.00*
**Strain53**	0.36 ± 0.04 vs. 1 ± 0.00*	0.37 ± 0.05 vs. 1 ± 0.00*
**Strain54**	0.37 ± 0.06 vs. 1 ± 0.00*	0.57 ± 0.03 vs. 1 ± 0.00*
**Strain60**	0.28 ± 0.00 vs 0.88 ± 0.00*	0.55 ± 0.04 vs. 1 ± 0.00*
**Strain61**	1 ± 0.00 vs. 1 ± 0.00	0.5 ± 0.03 vs. 1 ± 0.00*
**Strain70**	0.39 ± 0.05 vs. 1 ± 0.00*	0.76 ± 0.08 vs. 1 ± 0.00*
**Strain72**	0.30 ± 0.01 vs. 1 ± 0.00*	0.62 ± 0.02 vs. 1 ± 0.00*
**Strain82**	0.38 ± 0.05 vs. 1 ± 0.00*	0.50 ± 0.06 vs. 1 ± 0.00*
**Strain85**	0.40 ± 0.03 vs. 1 ± 0.00*	1 ± 0.00 vs. 1 ± 0.00
**Strain107**	1 ± 0.00 vs. 1 ± 0.00	0.54 ± 0.01 vs. 1 ± 0.00*
**Strain06A**	0.28 ± 0.02 vs. 1 ± 0.00*	1 ± 0.00 vs. 1 ± 0.00
**Strain06L**	0.45 ± 0.03 vs. 1 ± 0.00*	0.56 ± 0.00 vs. 1 ± 0.00*
**Strain10Li**	0.59 ± 0.03 vs. 1 ± 0.00*	0.52 ± 0.02 vs. 1 ± 0.00*
**Strain10S**	0.31 ± 0.04 vs. 1 ± 0.00*	0.79 ± 0.04 vs. 1 ± 0.00*
**Strain12A**	0.72 ± 0.05 vs. 1 ± 0.00*	0.50 ± 0.00 vs. 1 ± 0.00*
**Strain12L**	0.56 ± 0.02 vs. 1 ± 0.00*	0.55 ± 0.02 vs. 1 ± 0.00*
**Strain15A**	0.75 ± 0.00 vs. 1 ± 0.00*	0.51 ± 0.00 vs. 1 ± 0.00*
**Strain15G**	0.44 ± 0.07 vs. 1 ± 0.00*	0.54 ± 0.02 vs. 1 ± 0.00*
**Strain15L**	0.42 ± 0.02 vs. 1 ± 0.00*	0.47 ± 0.03 vs. 1 ± 0.00*
**Strain18A**	0.4 ± 0.00 vs. 1 ± 0.00*	0.52 ± 0.03 vs. 1 ± 0.00*
**Strain111L**	0.29 ± 0.01 vs. 1 ± 0.00*	0.67 ± 0.04 vs. 1 ± 0.00*
**Strain111R**	0.42 ± 0.02 vs. 1 ± 0.00*	0.68 ± 0.02 vs. 1 ± 0.00*

*P < 0.05, **CE: Crude Extract of *Eugenia uniflora* (2000μg/mL); † Pz = Precipitation zone.

The isolates were incubated in triplicates at 30°C for 72 h on proteinase or phospholipase agar in the absence (control experiments) or presence of 2000 μg/ml (test experiments) of the CE of *Eugenia uniflora*.

## Discussion

In this study we investigated a possible role of the CE of *E. uniflora* in some virulence factors of *C. albicans.* In a previous study performed by Ferreira et al. [43], a screening of antifungal activity of medicinal plants from northeastern Brazil, including 30 different vegetal CEs, revealed *E. uniflora* as the most active natural product, with minimal inhibitory concentration (MIC) values ranging from 31.25 to 62.5 μg/mL against *C. albicans*, as determined by broth microdilution methodology.

After the observation of *C. albicans* cells growth inhibition with the CE of *E. uniflora*, we decided to investigate a possible action of this natural product in the impairment of the full expression of some virulence factors in *C. albicans*. Therefore, we have used NGY broth to grow our strains in the presence of the CE previously to each attribute of virulence tested *in vitro*. We hypothesized that when growing our strains in the presence of this natural product, *C. albicans* cells would be impaired to fully express the pathogenicity factors investigated. Therefore a pilot experiment revealed that 2000 μg/ml of the *E. uniflora* CE was needed to inhibit approximately 50% growth of cells incubated for 18 to 24 h in NGY broth under 200 rpm, 30°C, but not to kill all of them. Of note, this is a complete different condition compared to the MIC determination experiment performed by Ferreira et al. [43], where the cells were incubated statically, in RPMI-1640, also in a different volume of culture medium. In our study, *C. albicans* cells were grown in NGY broth, 200 rpm. Incubation condition differences may explain discrepant MIC values within the two different methodologies.

When *C. albicans* cells were cultivated overnight in the presence of the natural product, bud cells demonstrated alterations in the cellular structure, suggesting a possible action of the CE of *E. uniflora* specifically on *C. albicans* cell wall.

It has been described that vegetal extracts can act on the cell wall of *C. albicans*. By analyzing the mechanism of action of the vegetal extract of *Stryphnodendron adstringens* on *C. albicans* cells, Ishida *et al.* [35] observed that the integrity of the cell wall was damaged, with the presence of several deformations in buddy cells structure.

It is important to emphasize that the recently developed antifungal drugs named echinocandins target *Candida* and *Aspergillus* cell wall, by inhibiting the enzyme β-1,3-D-glucan synthase [46]. These chemical compounds are less toxic than polyenes, which can also bind to mammalian cholesterol. Therefore, if *E. uniflora* compounds are truly acting on the cell wall, the natural product may be used for future development of antifungal drugs with lower side effects.

Bud-to-hyphae transition of *C. albicans* is a fundamental step for the establishment of infection, contributing to tissue invasion. We found that the ability to form true hyphae was reduced for the majority of isolates. Besides, all of the strains tested had a reduction in hyphal size in YPD + 20% FBS, when previously grown with the CE of *E. uniflora*. Of note, shorter true hyphae were not observed when *C. albicans* cells were incubated in liquid Spider medium and GlcNac liquid media, when previously grown in the presence of the CE. This may be explained because serum is the most potent inducer of hypha formation resulting in cells with higher MI [20]. Thus, due to the fact that the other two hyphal inducers already produced shorter true hyphae in *C. albicans*, a decrease in hyphal size could not be observed when cells were grown in the presence of the natural product.

Although others have found that natural products may impair morphogenesis [34,47,48], in our study we analyzed a higher number of strains and generated quantitative data of decreased hypha formation, under different bud-to-hyphae transition inducing media.

When *C. albicans* cells suspension were plated on the surface of Spider medium, GlcNac agar and YPD + 20% FBS agar, several phenotypic filamentous colonies were observed. When the CE of *E. uniflora* was added in the composition of the three media, it was observed a notorious reduction of filamentation of the colonies. Similar results were observed by Tsang et al*.* [34], who investigated the role of purpurin, a compound derived from *Rubia tinctorum* L. roots, in the impairment of filamentation of *C. albicans* on solid Spider medium*.*

Other important aspect of our findings is that the CE of *E. uniflora* clearly affects bud-to-hypha transition, observed under three different conditions. It is well known that morphogenesis in *C. albicans* can be triggered by the stimulation of several pathways or by essentially a single pathway, which is fully activated [49].

The secretion of hydrolytic enzymes such as proteinase and phospholipase are important virulence factors of *C. albicans,* contributing for the establishment of infection by damaging tissues and cellular membrane [25,50]. When the CE of *E. uniflora* was added to phospholipase and proteinase media, the zone of activity of the enzymes was either highly reduced or completely inhibited. This is an important finding because a reduction of production and secretion of these enzymes are related with a decrease of *C. albicans* virulence [51].

To the best of our knowledge, this is the first report in literature where the action of a natural product obtained from a plant is impairing the expression of both enzymes. The effect of the CE of *Dodonaea viscosa* var*. angustifolia* leaves, an indigenous South African plant*,* on proteinase and phospholipase activity in *C. albicans* was investigated in 26 strains isolated from the oral cavity of 150 HIV patients. Nevertheless, the secretion of both enzymes was not affected by the addition of the plant extract [52].

## Conclusions

This was the first study to deeply investigate the effect of the CE of *E. uniflora* in virulence factors of *C. albicans* clinical isolates collected in a specific clinical scenario (oral candidiasis in kidney transplant recipients). Our study has proved a satisfactory effect of this natural product in inhibiting three important virulence factors of *C. albicans*. We also verified that more than 80% of A549 cells (human alveolar basal epithelial cell line) remained viable even when exposed to 4 fold concentration of the CE of *E. uniflora* (8000 μg/ml; unpublished data), suggesting its possible safety if applied as an antifungal agent in the future. Our group is currently working on the elucidation of the chemical compounds responsible for the inhibition of growth and specifically interactions with virulence factors.

## Abbreviations

ATCC: American type culture collection
BSA: Bovine serum albumine
C.: *Candida*
CE: Crude extract
CO_2_: carbonic gas
°C: Grade celsius
DO: Optical density
et al.: Collaborators
FBS: Fetal bovine serum
g/l: Gram per liter
GlcNac: N-acetyl-D-glucosamine
h: Hour
MI: Morphological index
KCl: Sodium chloride
KH_2_PO_4_: Monobasic potassium phosphate
M: Molar
MIC: Minimal inhibitory concentration
min: Minute
MI: Morphology index
mL: Milliliter
mL/L: Milliliter per liter
mM: Milimolar
mm: Millimeter
MgCl_2_: Magnesium chloride
mg/mL: Milligram per milliliter
μg/mL: Microgram per milliliter
μL: Microliter
N°: Number
NaCl: Sodium chloride
Na_2_HPO_3_: Sodium phosphate dibasic
NGY: Neopeptone – yeast extract - glucose
nm: Nanometer
pH: Hydrogen potential
Pz: Precipitation zone
®: Trademark
Rpm: Revolutions per minute
Saps: Secreted aspartic proteinases
SDA: Sabouraud dextrose agar
spp.: Species
TM: Trade mark
UFRN: Universidade Federal of Rio Grande do Norte
YPD: Yeast peptone dextrose
YCB: Yeast carbon base
